# Biochemical Characterization of Novel Retroviral Integrase Proteins

**DOI:** 10.1371/journal.pone.0076638

**Published:** 2013-10-04

**Authors:** Allison Ballandras-Colas, Hema Naraharisetty, Xiang Li, Erik Serrao, Alan Engelman

**Affiliations:** Department of Cancer Immunology & AIDS, Dana-Farber Cancer Institute, and Department of Medicine, Harvard Medical School, Boston, Massachusetts, United States of America; Queensland Institute of Medical Research, Australia

## Abstract

Integrase is an essential retroviral enzyme, catalyzing the stable integration of reverse transcribed DNA into cellular DNA. Several aspects of the integration mechanism, including the length of host DNA sequence duplication flanking the integrated provirus, which can be from 4 to 6 bp, and the nucleotide preferences at the site of integration, are thought to cluster among the different retroviral genera. To date only the spumavirus prototype foamy virus integrase has provided diffractable crystals of integrase-DNA complexes, revealing unprecedented details on the molecular mechanisms of DNA integration. Here, we characterize five previously unstudied integrase proteins, including those derived from the alpharetrovirus lymphoproliferative disease virus (LPDV), betaretroviruses Jaagsiekte sheep retrovirus (JSRV), and mouse mammary tumor virus (MMTV), epsilonretrovirus walleye dermal sarcoma virus (WDSV), and gammaretrovirus reticuloendotheliosis virus strain A (Rev-A) to identify potential novel structural biology candidates. Integrase expressed in bacterial cells was analyzed for solubility, stability during purification, and, once purified, 3′ processing and DNA strand transfer activities *in vitro*. We show that while we were unable to extract or purify accountable amounts of WDSV, JRSV, or LPDV integrase, purified MMTV and Rev-A integrase each preferentially support the concerted integration of two viral DNA ends into target DNA. The sequencing of concerted Rev-A integration products indicates high fidelity cleavage of target DNA strands separated by 5 bp during integration, which contrasts with the 4 bp duplication generated by a separate gammaretrovirus, the Moloney murine leukemia virus (MLV). By comparing Rev-A *in vitro* integration sites to those generated by MLV in cells, we concordantly conclude that the spacing of target DNA cleavage is more evolutionarily flexible than are the target DNA base contacts made by integrase during integration. Given their desirable concerted DNA integration profiles, Rev-A and MMTV integrase proteins have been earmarked for structural biology studies.

## Introduction

Integrase (IN) is a key protein in the replicative cycle of retroviruses, integrating reverse-transcribed linear viral DNA (vDNA) into a chromosome of the infected host cell [1]. Retroviral integration proceeds in four steps: (i) IN-vDNA binding to form the stable synaptic complex or intasome, which is comprised of an IN tetramer and the U3 and U5 ends of vDNA, (ii) 3′ processing, (iii) DNA strand transfer, and (iv) DNA gap repair. IN 3′ processing activity in most cases cleaves two nucleotides from both the U3 and U5 vDNA ends, generating reactive CA_OH_-3′ end sequences. In the DNA strand transfer step, IN uses the CA_OH_-3′ ends to attack a pair of phosphodiester bonds that are separated on opposing chromosomal target DNA strands by four to six nucleotides, depending on the retrovirus. Gap repair of the concerted DNA strand transfer reaction product yields a duplication of 4–6 bp of target DNA flanking the integrated provirus.

Retroviral IN proteins consist of three domains: the zinc-binding N-terminal domain (NTD), the catalytic core domain that contains the invariant D,D(35)E enzyme active site catalytic triad, and the C-terminal domain (reviewed in [2]). Epsilonretrovirus, gammaretrovirus, and spumavirus INs differ from the other retroviral INs by the presence of a fourth domain, the N-terminal extension domain, which precedes the NTD [3].

Major obstacles in the structural biology of retroviral IN proteins include the propensity for protein aggregation under conditions of limited ionic strength and the presence of flexible linkers connecting the different protein domains [2]. Despite these limitations, X-ray crystal structures of the spumavirus prototype foamy virus (PFV) intasome that represent the salient nucleoprotein complexes along the first three steps in the integration pathway have been determined [4]–[6]. These advances are in large part due to favorable PFV IN biochemical properties, which include highly soluble protein and the ability to efficiently integrate two surrogate vDNA ends in concerted fashion into target DNA *in vitro* [7], [8]. The intasome structures provide unprecedented details on the molecular mechanism of retroviral DNA integration as well as the mechanisms of action of clinical strand transfer inhibitors [4]–[6], [9]. To date, only the PFV IN has yielded diffractable intasome crystals. Our long-term goal is to increase the repertoire of retroviral intasome structures. Toward this end, we have characterized five previously unstudied retroviral IN proteins.

The INs from the alpharetrovirus lymphoproliferative disease virus (LPDV) [10], betaretroviruses Jaagsiekte sheep retrovirus (JSRV) [11] and mouse mammary tumor virus (MMTV) [12], epsilonretrovirus walleye dermal sarcoma virus (WDSV) [13], and gammaretrovirus reticuloendotheliosis virus strain A (Rev-A) [14] were expressed as hexahistidine (His_6_) fusion proteins in bacteria. Here we examine the solubility of the proteins, their stability during purification, and the ability for the purified proteins to support IN activities *in vitro* under a variety of reaction conditions. Our results show that WDSV IN is insoluble under the tested conditions while JRSV and LPDV INs were unstable and precipitated during protein purification. Purified, active MMTV and Rev-A INs were by contrast obtained from bacterial cell lysates. We show that both enzymes preferentially integrate two vDNA ends under concerted integration reaction conditions. Interestingly, our data reveal that gammaretroviral Rev-A and MLV INs show very similar base preferences at the sites of integration despite generating different lengths of duplicated target DNA sequence.

## Materials and Methods

### Bacterial expression vectors and oligonucleotides

Most IN proteins were expressed from the pFVmarIN derivative of bovine immunodeficiency virus (BIV) IN expression vector pCPH6P-BIV-IN [15], which directs the synthesis of N-terminal His_6_-tagged proteins followed by a cleavage site for human rhinovirus (HRV) 3C protease; LPDV IN was by contrast expressed from pRSET-A (Life Technologies, Grand Island, NY) as a His_6_ fusion protein. The following DNAs were used as PCR templates: JSRV, pCMV2JS21 [16]; WDSV, pDL1 [17]; MMTV, pMMTV-HP [12]; Rev-A, pSW253 [14]. The JSRV IN sequence amplified by PCR using primers AE4484 and AE4485 (see Table 1 for a list of oligonucleotides used in this study) was cleaved with XmaI and BglII, and the cut DNA was ligated to XmaI/BamHI-digested pFVmarIN; WDSV and MMTV IN sequences were similarly introduced into the pFVmarIN backbone. The Rev-A IN sequence amplified using primers AE4506 and AE4507 was cleaved with NdeI and BglII and ligated with NdeI/BamHI-digested pFVmarIN. The LPDV IN reading frame, which was synthesized de novo (Life Technologies), was cut with BamHI and XhoI, and then ligated to BamHI/XhoI-digested pRSET-A. The sequences of IN reading frames were verified by dideoxy sequencing.

**Table 1:** Oligonucleotides used in this study.

Name	Sequence	Use	Reference
AE191	5′-TTTTAGTCAGTGTGGAAAATCTCTAGCAG	HIV-1 U5 end –T (29-mer)	[19]
AE143	5′-ACTGCTAGAGATTTTCCACACTGACTAAAA	HIV-1 U5 minus strand (30-mer)	[19]
AE3652	5′- ACTGCTAGAGATTTTCCACACTGACTAAAAGG	HIV-1 U5 minus strand (32-mer)	[19]
AE3653	5′-CCTTTTAGTCAGTGTGGAAAATCTCTAGCA	HIV-1 U5 end precleaved (30-mer)	[19]
AE3715	5′-GTGATATTGCTGAAGAGCTTG	Integration site sequencing primer 1	This study
AE3717	5′-ATTCCACAGCTGGTTCTTTC	Integration site sequencing primer 2	This study
AE4468	5′-ATTGTCATGGAATTTTGTATATTGATTATCCT	PFV U5 end minus strand (EV54)	[8]
AE4469	5′-AGGATAATCAATATACAAAATTCCATGACA	PFV U5 end preprocessed (EV55)	[8]
AE4474	5′-CAGGGACCCGGGGTACTCAAGAAGGGGGACGCC	WDSV IN PCR primer1	This study
AE4475	5′-GCGCGCAGATCTTAAGAAAGTAGCTGGTTGACTGG	WDSV IN PCR primer2	This study
AE4484	5′-CAGGGACCCGGGTCAGCTATTGATGCAGCCCGG	JSRV IN PCR primer1	This study
AE4485	5′-GCGCGCAGATCTCACTCGTGGGCTCGCTCAGC	JSRV IN PCR primer2	This study
AE4494	5′-CAGGGACCCGGGGCTTTAGAGTCAGCTCAAGAAAGC	MMTV PCR primer1	This study
AE4495	5′-GCGCGCAGATCTTAAGGACCTCCTCCGCTTCGG	MMTV PCR primer 2	This study
AE4503	5′-CGGTGACCCTCAGGTCGGCCGACTGCGGCAT	MMTV U5 end –T (31-mer)	This study
AE4504	5′-CGGTGACCCTCAGGTCGGCCGACTGCGGCA	MMTV U5 end precleaved (30-mer)	This study
AE4505	5′-AATGCCGCAGTCGGCCGACCTGAGGGTCACCG	MMTV U5 end minus strand (32-mer)	This study
AE4506	5′-GCGCGCCATATGCTTGAAGTCCTCTTTCAGGGACCCGATGCACCGGATATGCCAGATACC	Rev-A IN PCR primer1	This study
AE4507	5′-GCGCGCAGATCTTAGGATTTTGCTCGCCTGGTCAAC	Rev-A IN PCR primer2	This study
AE4514	5′-GGACTGAATCCGTAGTACTTCGGTACAACAT	Rev-A U5 end –T (31-mer)	This study
AE4515	5′-GGACTGAATCCGTAGTACTTCGGTACAACA	Rev-A U5 end precleaved (30-mer)	This study
AE4516	5′-AATGTTGTACCGAAGTACTACGGATTCAGTCC	Rev-A U5 end minus strand (32-mer)	This study
AE5193	phospho-5′-CAGGGCGCGTCAGGTGGCACT	pEGFP-C1 PCR primer1	This study
AE5194	phospho-5′-TTTCATAGAAGGCGGCGGTGG	pEGFP-C1 PCR primer2	This study

### Protein expression and purification

*Escherichia coli* strain PC2 [15] carrying the various IN expression constructs was grown in LB broth in the presence of 40 µM ZnSO_4_. Optimal expression conditions based on the temperature (18°C, 25°C, 30°C, or 37°C) and time (4 h, 6 h, or 12 h) of induction, as well as the concentration (0.1 mM, 0.3 mM, 0.5 mM, or 1 mM) of the chemical isopropyl-β-_D_-thiogalactopyranoside (IPTG) inducer, were independently established for each IN.

Induced bacterial cultures were harvested by centrifugation at 6,000 X *g*, and pellets were dissolved in buffer A (20 mM HEPES, pH 7.6, 200 mM NaCl, 1 mM phenylmethanesulfonylfluoride [PMSF]). Following sonication for 1.5 min at 50 mA, the cell lysate was centrifuged at 60,000 X *g* for 45 min. The resulting S1 supernatant fraction was saved, and the P1 pellet was resuspended in buffer B (20 mM HEPES, pH 7.6, 1 M NaCl, 5 mM 3-{[3-cholamidopropyl] dimethylammonio}-2-hydroxy-1-propanesulfonate [CHAPS], 1 mM PMSF) by homogenization. Supernatant fractionation S2 was saved following centrifugation, while pellet P2 was resuspended by homogenization in buffer C (20 mM HEPES, pH 7.6, 0.5 M NaCl, 2 M urea, 1 mM PMSF). Final S3 and P3 fractions were made after centrifugation. Fractions were analyzed by western blot using anti-His_6_ monoclonal antibody conjugated to horseradish peroxidase (Clontech, Mountain View, CA) at 1∶10,000 dilution.

LPDV and JSRV IN S1 fractions were filtered through 0.45 µm filters, and the filtrates were loaded onto Ni^2+^-charged HisTrap Chelating HP columns (GE Healthcare, Pittsburgh, PA) previously equilibrated in buffer A supplemented to contain 5 mM imidazole. Proteins were eluted with a linear gradient of imidazole from 5 mM to 500 mM using an ÄKTA chromatography system (GE Healthcare). IN-containing fractions, which were identified following sodium dodecyl sulfate–polyacrylamide gel electrophoresis (SDS-PAGE) and staining with Coomassie blue, were dialyzed overnight against 20 mM HEPES, pH 7.6, 50 mM NaCl, 1 mM EDTA, 5 mM dithiothreitol (DTT) at 4°C. Dialysates were then either loaded onto a HiTrap ANX anion exchange column (GE Healthcare) or a HiTrap SP HP cation exchange column (GE Healthcare), each pre-equilibrated with 20 mM HEPES, pH 7.6, 50 mM NaCl. INs were eluted from ion exchange columns using a linear gradient of NaCl from 50 mM to 1 M. Fractions were detected as above.

LPDV and JSRV INs from fraction S3 were filtered through 0.45 µm filters, and filtrates were loaded onto HisTrap Chelating HP columns pre-equilibrated with 20 mM HEPES pH 7.6, 200 mM NaCl, 8 M urea, 5 mM imidazole. Columns were washed with 10 column volumes of buffer C adjusted to contain 8 M urea and 30 mM imidazole, and the INs were eluted by a linear gradient of imidazole from 30 mM to 0.5 M. The INs were refolded using one of three techniques: (i) rapid dilution (1∶10) in ice cold 20 mM HEPES, pH 7.6, 200 mM NaCl, 5 mM DTT, (ii) successive dialysis to remove urea against: (a) 20 mM HEPES, pH 7.6, 200 mM NaCl, 2 M urea, 10 mM CHAPS, 5 mM DTT, 1 mM EDTA, (b) 20 mM HEPES, pH 7.6, 200 mM NaCl, 1 M urea, 10 mM CHAPS, 5 mM DTT, 1 mM EDTA, and (c) 20 mM HEPES, pH 7.6, 500 mM NaCl, 10 mM CHAPS, 5 mM DTT, or (iii) directly on the column by gradient reduction of urea from 8 to 0 M.

MMTV and Rev-A INs in fraction S2 were filtered through 0.45 µm filters, and filtrates were loaded onto Ni^2+^-charged HisTrap Columns previously equilibrated with buffer B–20 mM imidazole. Proteins were eluted by a linear gradient of imidazole from 20 mM to 500 mM using the ÄKTA purifier system. IN containing fractions, which were identified by Coomassie blue staining after SDS-PAGE, were dialyzed against buffer B, and the His_6_ tag was removed by cleavage with HRV 3C protease (GE Healthcare) overnight at 4°C, yielding protein N-termini containing the heterologous Gly-Pro sequence. Cleaved MMTV and Rev-A INs were purified by gel filtration on a Superdex 200 column respectively equilibrated with buffer D (25 mM Tris-HCl, pH 7.4, 0.5 M NaCl, 5 mM CHAPS, 2 mM DTT) and buffer E (20 mM HEPES, pH 7.6, 1 M NaCl, 2 mM CHAPS, 2 mM DTT). Purified INs were concentrated by ultrafiltration using 10-kDa molecular weight cutoff Millipore concentrators, and retentates were dialyzed overnight against buffer D or buffer E, each supplemented to contain 10% glycerol. Protein concentration was determined by spectrophotometry, and aliquots flash-frozen in liquid N_2_ were stored at –80°C. Protein purity was quantified by analyzing silver-stained SDS-polyacrylamide gels using Molecular Imager® Gel Doc™ XR+ System and Image Lab software (Bio-Rad, Hercules, CA). The multimeric state of purified Rev-A and MMTV IN proteins (200 µg) was analyzed by gel filtration chromatography using a HiLoad 26/60 Superdex 200 column equilibrated in 20 mM HEPES, pH 7.6, 1 M NaCl, 7.5 mM CHAPS, 2 mM DTT.

Recombinant lens epithelium-derived growth factor (LEDGF)/p75 [18] and human immunodeficiency virus type 1 (HIV-1) [19] and PFV [8] IN proteins expressed in bacteria were purified as previously described.

### IN 3′ processing activity assay

3′ Processing substrates mimicked the U5 DNA ends of various retroviruses. Oligonucleotide pairs AE4503/AE4505, AE4514/AE4516, and AE191/AE143 represented MMTV, Rev-A, and HIV-1 vDNAs, respectively, with one nucleotide omitted from the 3′ ends of the transferred DNA strands. DNA duplexes, which were annealed by heating for 3 min at 85°C in 100 mM NaCl, were filled in with [α-^32^P]TTP (3,000 Ci/mmol; PerkinElmer, Waltham, MA) using Sequenase version 2.0 T7 DNA polymerase (GE Healthcare) [20]. Unincorporated radionucleotide was removed by passing mixtures through Bio-Spin 6 columns (Bio-Rad) equilibrated with 10 mM Tris-HCl, pH 8.0, 20 mM NaCl, 0.1 mM EDTA.

IN (0.5 µM) was incubated with 15 nM labeled DNA for 1 h at 37°C in 25 mM MOPS, pH 7.2, 10 mM DTT, 5 µM ZnSO_4_, 10 mM MgCl_2_ or MnCl_2_, with or without 10% glycerol in 20 µL. The reaction was stopped by addition of 20 µL of sequencing gel sample buffer (95% formamide, 10 mM EDTA, 0.003% xylene cyanol, 0.003% bromophenol blue) and boiling for 2 min. DNA (1 µL) was fractionated through denaturing 20% polyacrylamide gels, and products visualized using a Storm 820 PhosphorImager were quantified by ImageQuant version 1.2 (GE Healthcare).

### DNA strand transfer activity assay

Substrates that mimicked preprocessed MMTV, Rev-A, HIV-1, and PFV U5 vDNA ends for DNA strand transfer activity assays were prepared by 5′ end-labeling oligonucleotides AE4504, AE4515, AE3653, and AE4468 with [γ-^32^P]ATP (3,000Ci/mmol; PerkinElmer) using Optikinase (Affymetrix, Santa Clara, CA) and annealing unlabeled strands AE4505, AE4516, AE3652, and AE4469, respectively. Unincorporated radionucleotide was removed by passing the annealed vDNAs through Bio-Spin 6 columns as above.

IN (0.8 µM) was mixed with 0.5 µM vDNA, of which 5% was 5′ end labeled, and 0.3 µg pGEM-3 target DNA in 20 mM HEPES, pH 7.4, 32 mM NaCl, 5 mM MgCl_2_, 4 µM ZnSO_4_, 10 mM DTT in 40 µL. After 15 min at room temperature, 0.6 µM LEDGF/p75 was added to the HIV-1 IN-containing reaction, then mixtures were incubated for 1 h at 37°C; reactions were stopped by adding 25 mM EDTA–0.5% SDS. Products deproteinized by digestion with proteinase K and precipitated with ethanol were analyzed by electrophoresis through 1.5% agarose gels, and DNAs were visualized using ethidium bromide (EtBr) staining. After drying, radiolabeled DNA was visualized using a Storm 820 PhosphorImager.

### Sequence analysis of Rev-A integration products

Integration products were cloned and sequenced essentially as described previously for BIV and equine infectious anemia virus IN [15]. Briefly, the strand transfer assay for Rev-A IN was scaled up 30-fold using the unlabeled preprocessed vDNA substrate. Integration products were separated on 1.5% agarose gels, and linear DNA consistent with the concerted integration of two vDNA ends was extracted using the Qiagen Gel Extraction kit; DNA was eluted in 50 µL H_2_O. DNA precipitation by ethanol in the presence of GenElute Linear PolyAcrylamide (Sigma-Aldrich, St. Louis, MO) was resuspended in 10 µL H_2_O, treated with Phi29 DNA polymerase (New England Biolabs, Beverly, MA) in the presence of 200 µM dNTP, 50 mM Tris-HCl, pH 7.5, 10 mM MgCl_2_, 10 mM (NH_4_)_2_SO_4_, and 4 mM DTT, and 5′-phosphorylated using Optikinase in the presence of 1 mM ATP, 50 mM Tris-HCl, pH 7.5, 10 mM MgCl_2_, 5 mM DTT. A kanamycin-resistance cassette prepared by PCR amplification of pEGFP-C1 (Clontech, Mountain View, CA) using primers AE5193 and AE5194 was digested with Dpn I to digest the plasmid PCR template. DNA-repaired linear integration products were ligated to the kanamycin-resistance cassette; *E. coli* DH5a cells transformed with the ligation product were selected on agar plates containing 35 µg/mL kanamycin. Plasmids extracted from isolated colonies were sequenced using primers AE3715 and AE3717.

## Results

### Experimental strategy

The utility of PFV IN in retroviral structural biology [4]–[6] can be attributed to the solubility of the protein under conditions of limited ionic strength [7], [8] and the high efficiency of two-ended concerted vDNA integration into target DNA *in vitro* [4], [8]. Although the PFV intasome structures provide unprecedented details on the mechanism of retroviral DNA integration, they are based on a viral protein from a single genus of *Retroviridae*. Our long-term goal is to expand the repertoire of retroviral intasome structures and, toward this end, we have expressed five previously uncharacterized IN proteins from four of the other six viral genera in the hope of finding molecules with desirable protein solubility and/or concerted integration activity profiles. The five proteins are derived from the alpharetrovirus LPDV, betaretroviruses MMTV and JSRV, epsilonretrovirus WDSV, and gammaretrovirus Rev-A.

### IN expression, solubility, and purification

IN proteins were engineered to be expressed as N-terminal His_6_ fusion proteins under the control of IPTG induction in bacteria. The temperature and time of induction, as well as IPTG concentration, was optimized for each expression construct, yielding the following parameters: WDSV IN, 18°C, 16 h, 0.5 mM IPTG; LPDV IN, 18°C, 4 h, 0.3 mM IPTG; JSRV IN, 18°C, 4 h, 0.3 mM IPTG; MMTV IN, 37°C, 6 h, 0.5 mM IPTG; Rev-A IN, 30°C, 16 h, 1 mM IPTG.

**Figure 1:**
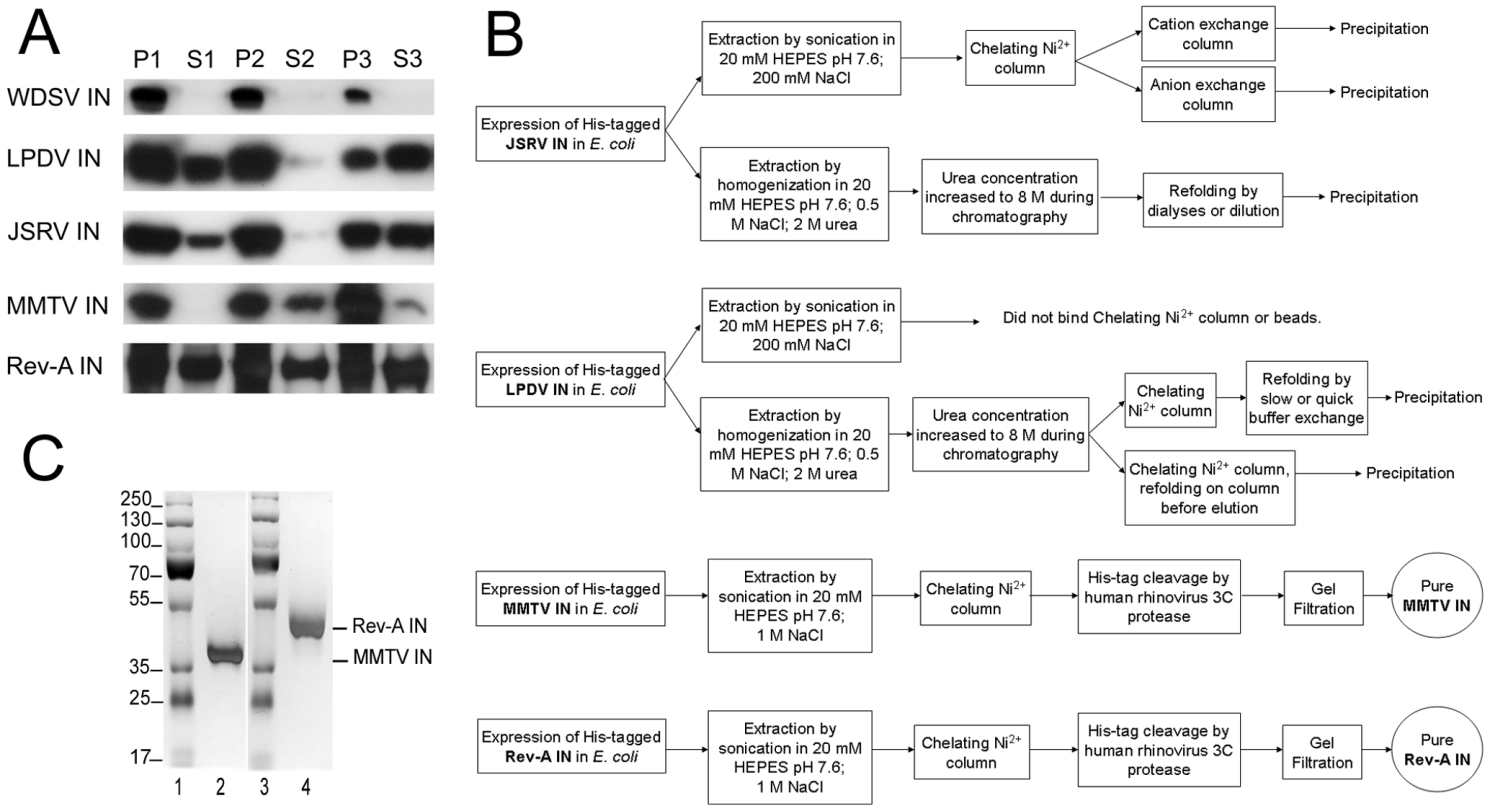
IN expression, extraction, and purification. (A) Fractions of bacterially expressed His_6_-tagged WDSV, LPDV, JSRV, MMTV, and Rev-A INs were visualized through western blotting. Lanes 1 and 2 represent the pellet (P1) and supernatant (S1) fractions obtained following centrifugation of cells lysed in 200 mM NaCl-containing buffer A. Pellet 2 (P2) and supernatant 2 (S2) were obtained following centrifugation (lanes 3 and 4) of fraction P1 homogenized in buffer B containing 1 M NaCl and 5 mM CHAPS. During the final extraction step, the pellet from step 2 was homogenized in buffer C containing 0.5 M NaCl and 2 M urea (lanes 5 and 6). (B) Schematic of the protocols utilized for JSRV, LPDV, MMTV, and Rev-A IN purification. All columns were run on an ÄKTA purifier system. (C) The purities of MMTV (lane 2) and Rev-A (lane 4) INs were assessed at 93% and 97%, respectively, following silver staining of SDS-polyacrylamide gels. Lanes 1 and 3 contain the indicated molecular mass standards.

The inherent solubility of each IN protein in *E. coli* extracts was assessed by lysing the cells by sonication in buffer containing relatively low salt concentration (200 mM NaCl), followed by extraction in two subsequent buffers which contained reagents to increasingly encourage protein solubilization (1 M NaCl–5 mM CHAPS, followed by 0.5 M NaCl–2 M urea). This strategy accordingly yielded six total fractions, three supernatants and three derived from the pellets after centrifugation (Fig. 1A). Visualization of SDS-polyacrylamide gels by western blotting revealed that a fraction of expressed LPDV, JSRV, and Rev-A proteins was solubilized following bacterial lysis in 200 mM NaCl-containing buffer A, as some of each appeared in fraction S1. By contrast, WDSV partitioned only to the pellet fractions (Fig. 1A). This largely insoluble protein was not investigated further.

Several strategies were tested to purify the LPDV and JSRV INs (Fig. 1B). Proteins extracted in buffer A were loaded onto Ni^2+^-charged HisTrap Chelating HP columns, and the proteins were eluted using a linear gradient of imidazole. Imidazole was subsequently removed by dialysis, and the INs were loaded onto anion or cation exchange columns, followed by elution using a linear gradient of NaCl. JSRV IN was observed to precipitate during this process, whereas LPDV IN did not effectively bind to the initial Ni^2+^-chelating column. In an attempt to bypass protein precipitation and to increase the yield of extracted protein, JSRV IN extracted from pellet P2 in 2 M urea-containing buffer C was purified by Ni^2+^-affinity chromatography in the presence of 8 M urea and refolded either by dialysis or by rapid dilution, which in both cases led to protein precipitation. LPDV IN from the supernatant S3 (buffer C) fraction was similarly utilized to promote binding to the Ni^2+^-affinity substrate. LPDV IN was subsequently refolded directly on the column by decreasing the concentration of urea in linear fashion, or after elution either by dialysis or by rapid dilution. In all cases, LPDV IN precipitated out of solution. Attempts to recover precipitated LPDV and JSRV IN proteins by resuspension in buffer C were unsuccessful.

MMTV and Rev-A INs extracted in 1 M NaCl-containing buffer B were loaded onto Ni^2+^-chelating columns and eluted using linear gradients of imidazole. Excess imidazole was removed by dialysis, and the His_6_ tag was removed by cleavage with the HRV 3C protease. MMTV and Rev-A INs were further purified by gel filtration chromatography, and then concentrated by ultrafiltration (Fig. 1B).

Highly purified (97%) Rev-A IN (2 mg) was recovered from 2 L of *E. coli* culture (1 mg/6 g of bacteria), while about 4 mg of MMTV IN (93% pure) was recovered from 4 L of culture (1 mg/4 g of bacteria) (Fig. 1C). Gel filtration analysis of the concentrated preparations revealed that Rev-A and MMTV INs migrated predominantly as monomers, with secondary species consistent with dimeric IN protein (Fig. S1).

### 3′ Processing of vDNA ends

**Figure 2:**
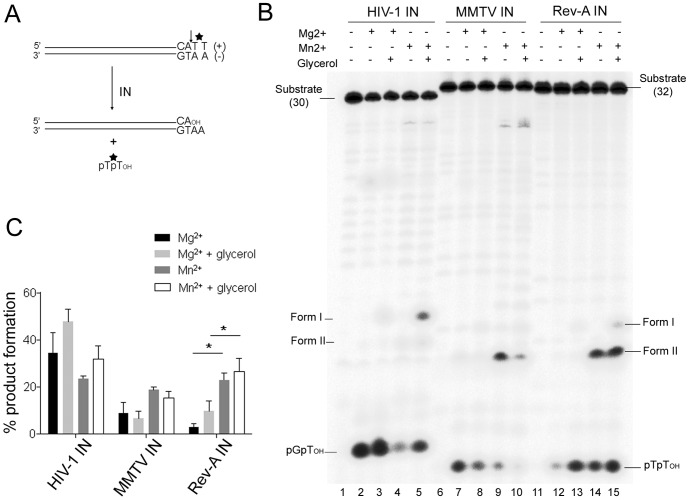
IN 3′ processing activities. (A) Schematic of blunt-ended vDNA substrate processed by IN adjacent to the conserved CA 3′ dinucleotide (vertical arrowhead). Positions of ^32^P label are shown by *. (B) Polyacrylamide sequencing gel of products of HIV-1, MMTV, and Rev-A IN 3′ processing reactions; Mn^2+^, Mg^2+^, and glycerol were included as indicated. The positions of the starting substrates (30 bp for HIV-1 IN; 32 bp for MMTV and Rev-A), the simple dinucleotide cleavage products (pGpT_OH_ for HIV-1 and pTpT_OH_ for MMTV and Rev-A), and form I and form II cleavage products are indicated. IN proteins were omitted from the initial reaction in each set of five reactions. (C) Mn^2+^ and Mg^2+^-dependent 3′ processing activities expressed as percentage of product formation ± standard error of the mean (SEM) for three independent experiments. Asterisks indicate *P* values <0.05 by paired t-test.

IN activity requires divalent metal ion cofactor, such as Mg^2+^ or Mn^2+^ [21], [22]. The activities of purified Rev-A and MMTV IN proteins were assessed under different reaction conditions. Initially, 3′ processing activity was measured using duplex oligonucleotides labeled within the dinucleotide that is cleaved by the IN protein (Fig. 2A). The 3′ ends of the vDNA plus-strands of MMTV and Rev-A terminate in TT, so 3′ processing of the DNA ends accordingly releases labeled pTT_OH_ cleavage products, which are readily assessed following denaturing polyacrylamide electrophoresis (Fig. 2B). As the 3′ end of the HIV-1 plus-strand terminates GT, pGT_OH_ is produced in this reaction.

In the presence of either Mg^2+^ or Mn^2+^ cofactor, MMTV and Rev-A INs processed their respective vDNA substrates (Fig. 2B, lanes 6–10 and 11–15). HIV-1 IN, used as a control, similarly processed its substrate (Fig. 2B, lanes 1–5). As previously established [20], HIV-1 IN generated the 3′–5′ cyclic dinucleotide product, also referred to as form II product, in the presence of Mn^2+^ (Fig. 2B, barely detected at this exposure level, lanes 4 and 5). Glycerol can also be used as an alternative nucleophilic agent instead of water, leading to formation of a glycerol dinucleotide adduct (also called form I cleavage product), and the alcoholysis pathway is likewise stimulated by Mn^2+^ [23]. Whereas the form I product dominated over form II in Mn^2+^-dependent reaction conditions with HIV-1 IN [20], Mn^2+^ preferentially stimulated the formation of form II over form I for both MMTV and Rev-A INs (Fig. 2B, lanes 4, 5, 9, 10, 14, and 15). We note a similar preference for the formation of the form II cyclic cleavage product in Mn^2+^-dependent 3′ processing reactions with the gammaretroviral IN protein from Moloney murine leukemia virus (MLV) [24].

HIV-1 IN 3′ processing activity was more efficient than either MMTV or Rev-A IN in the presence of Mg^2+^ (34% of the HIV-1 substrate processed in the absence of glycerol, compared to 9% and 3% for MMTV and Rev-A, respectively). Whereas glycerol afforded the altered choice of nucleophile in the presence of Mn^2+^, it did not significantly stimulate the overall extent of substrate processing under any condition tested. By contrast, Rev-A 3′ processing activity was stimulated significantly by Mn^2+^ (Fig. 2C).

### DNA strand transfer activity

**Figure 3:**
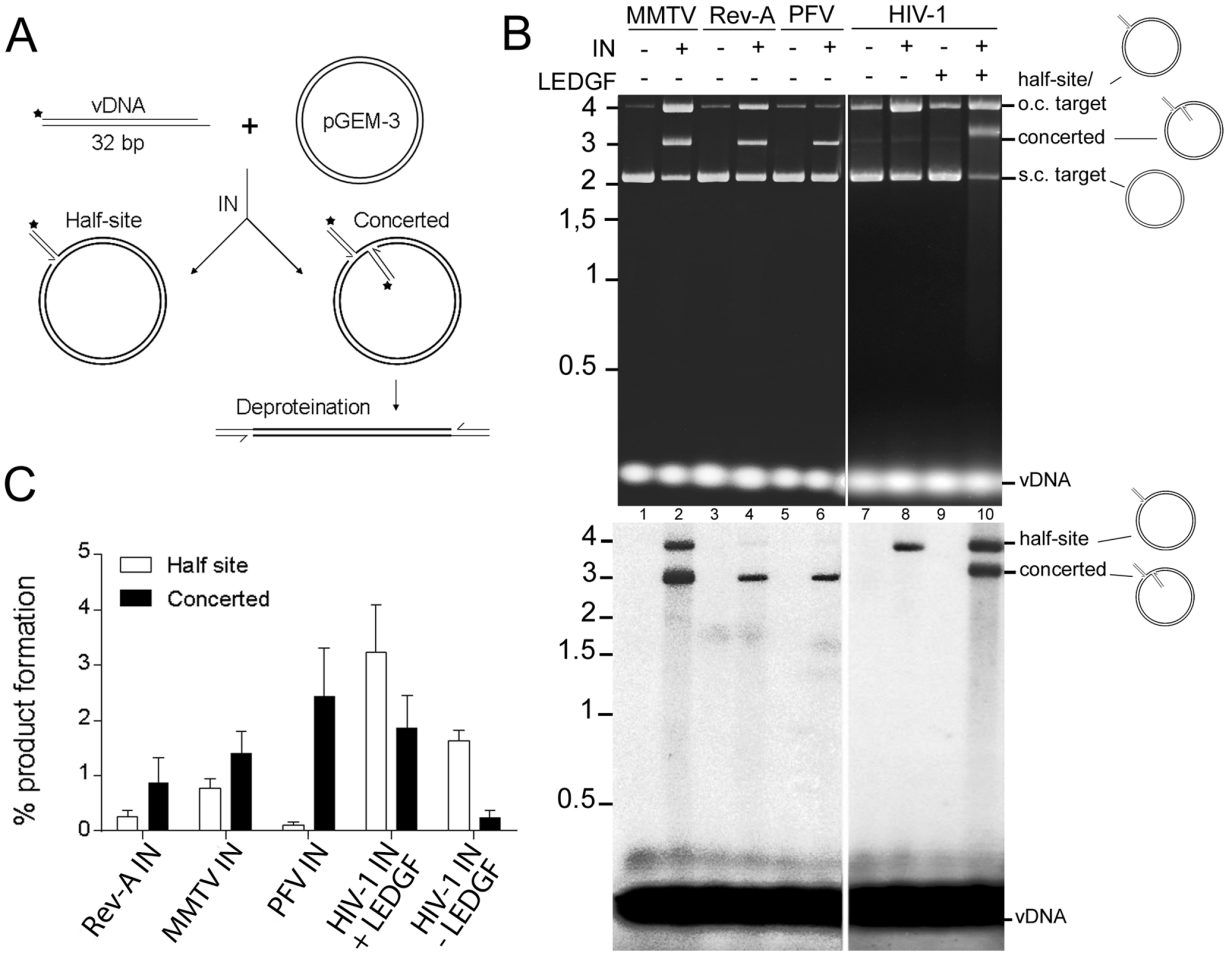
Concerted integration assay design and IN activities. (A) Schematic showing precleaved U5 substrate (vDNA), circular plasmid target DNA (pGEM-3), and products of single-end versus concerted vDNA integration. Positions of ^32^P label are shown by *. (B) EtBr stained image (upper panel) and phosphorimage (lower panel) of integration reactions, comparing MMTV and Rev-A INs to control PFV and HIV-1 IN proteins. Reactions fractionated through two separate gels delimitated by a white border were performed under the exact same conditions. Half-site products of Rev-A and PFV vDNA integration were evident upon long exposure of the phosphorImager screen. Migration positions of standards (in kb) are shown to the left, whereas positions of half-site and concerted vDNA integration products are to the right. Note the half-site products co-migrate with the open circular (o.c.) form of pGEM-3, whereas the concerted products migrate in between the o.c. and supercoiled (s.c.) forms of the plasmid. (C) Half-site and concerted integration quantification of panel B phosphorimage. Results (percent of vDNA substrate converted into half-site and concerted integration reaction products) are means ± SEM for three independent experiments.

The DNA strand transfer activity assay was designed to monitor the extent of concerted vDNA integration in addition to the integration of single vDNA ends into target DNA (Fig. 3A). Pre-processed, 5′-end labeled DNAs that mimic the U5 ends of the various viruses were incubated with pGEM-3 circular plasmid DNA as the integration target. The integration of a single vDNA end into one strand of target DNA yields a tagged circular product that co-migrates with the open circular plasmid DNA molecule, whereas concerted integration yields a linear product that migrates close to the linearized form of the plasmid (∼3 kb) (Fig. 3A). HIV-1 IN and PFV IN were used as positive controls. As expected, HIV-1 IN yielded only half-site integration products in the absence of additional protein co-factors (Fig. 3B, lane 8); the addition of the LEDGF/p75 co-factor increased the overall extent of IN activity, and significantly stimulated the formation of concerted vDNA integration products (Fig. 3B, lane 10) [25]. PFV IN, also as expected, generated a predominance of concerted integration products in the absence of IN-binding co-factors (Fig. 3B, lane 6) [4], [8]. MMTV and Rev-A INs also preferentially catalyzed the concerted integration of two vDNA ends over the half site integration of a single vDNA into target DNA (Fig. 3B, lanes 2 and 4; exemplified in the lower phosphorImager panel). Under these reaction conditions, MMTV IN converted about 1.4% and 0.8% of the substrate into concerted and half-site integration products, respectively, while ∼0.9% and 0.3% were generated by Rev-A IN (Fig. 3C).

### Sequence analysis of Rev-A concerted integration products

Both MMTV and Rev-A INs catalyzed 3′ processing and concerted vDNA strand transfer activities, and thus could be good structural biology candidates. The integration site preferences of MMTV have been analyzed extensively in virus-infected cells [26], whereas only a handful (8 total) of integration sites have been reported for spleen necrosis virus (SNV) [27], [28], an avian gammaretrovirus that is closely related to Rev-A [29]. Because the limited number of proviruses precluded the assessment of nucleotide preferences at the sites of SNV integration in cells [28], we cloned and sequenced products of *in vitro* concerted Rev-A DNA integration reactions to more fully characterize the integration mechanism of this species of gammaretrovirus. The linear DNA products, which were treated with the strand-displacing Phi29 DNA polymerase and phosphorylated, were ligated to a blunt-ended kanamycin resistance cassette prior to transformation of *E. coli* cells. Plasmids extracted from isolated colonies were sequenced using outward facing primers that annealed to the flanking regions of the kanamycin cassette; Table 2 summarizes the different types of integration products obtained. Although a significant number of recovered DNA products contained only a single viral DNA end, all products of concerted vDNA integration notably harbored a duplication of 5 bp of target DNA sequence.

**Table 2:** Rev-A integration products obtained by DNA sequence analysis.

Type of integration product	Number of colonies
Concerted integration with 5 bp duplication	26
Concerted integration with duplication other than 5 bp	<1
Half-site integration	13
Multiple half-site integrations	4

**Figure 4:**
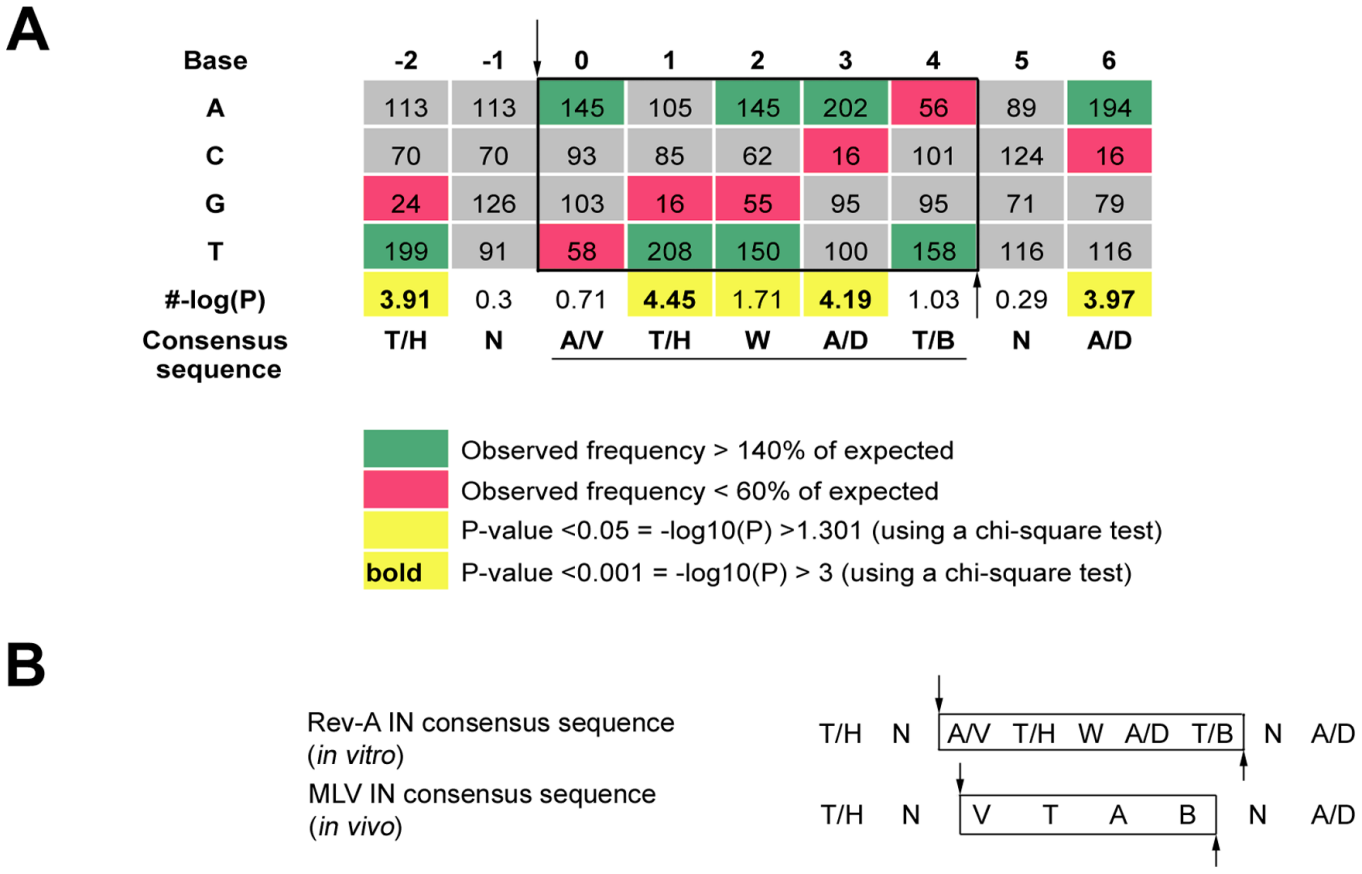
Sequence analysis of Rev-A integration sites and comparison to MLV. (A) Palindromic consensus sequence from sites of Rev-A integration *in vitro*. Observed frequencies of nucleotides at the insertion sites were compared to expected frequencies at each position based on the sequence of the pGEM-3 target DNA. The sequence of the target site duplication following DNA gap repair is indicated in the black box and underlined below the consensus sequence, which employs IUPAC-IUB nucleotide codes; positions of DNA strand transfer are labeled by vertical arrows. Green and red boxes highlight nucleotide positions that are >140% and <60% of the expected base, respectively. Yellow boxes and bold values indicate *P* values of <0.05 and 0.001, respectively. (B) Comparison of consensus Rev-A (from panel A) and MLV [36] integration site sequences.

Target DNA nucleotides in the immediate vicinity of the Rev-A concerted DNA integration sites were analyzed by comparing the observed frequencies to the expected frequencies at each position based on the sequence of the pGEM-3 target DNA, which is 24.8% A, 25.8% C, 25.3% G, and 24.1% T (Fig. 4A). Using the nomenclature recommended by the International Union of Pure and Applied Chemistry-International Union of Biochemistry (IUPAC-IUB) [30], the following consensus sequence was ascribed: (T/H)N↓(A/V)(T/H)W(A/D)(T/B)N(A/D) (the arrow indicates the position of plus-strand joining; the underline indicates the sequence of 5 bp duplication).

## Discussion

In this study five previously uncharacterized retroviral IN proteins were analyzed following their expression in bacteria. Our long-term goal is generating 3-dimensional structures of retroviral intasome complexes. Toward this end, the goal of this study was to characterize the inherent solubilities and concerted integration activities of the novel IN proteins.

Our results show that epsilonretrovirus WDSV IN is not soluble under the tested conditions, which included induction of protein expression in *E. coli* cultures propagated at 18°C. Epsilonretrovirus is the only retroviral genus for which an IN protein has not previously been characterized. It has been suggested that WDSV reverse transcriptase is temperature sensitive, displaying optimal activity at 4–15°C, which might reflect the natural habitat of the fish host [31]. We can hypothesize that WDSV IN might also be temperature sensitive, and that our tested conditions did not satisfy its parameters for proper folding when expressed in bacteria.

We were able to express and extract alpharetrovirus LPDV IN, betaretrovirus JSRV IN and MMTV IN, and gammaretrovirus Rev-A IN. However, despite extensive effort (Fig. 1B), we were unable to purify accountable amounts of either LPDV or JSRV IN. It could be useful to test different expression systems, for example baculovirus in insect cells, to see if the biophysical properties of these INs might improve during purification. Due to our long-term goal, we have focused in this study on activity characterization of proteins that could be recovered from bacterial extracts. By monitoring 3′ processing and DNA strand transfer activities, we show that MMTV and Rev-A INs each preferentially catalyze the concerted integration of two vDNA ends into target DNA under conditions, that in the absence of the LEDGF/p75 co-factor, favored HIV-1 IN half-site integration activity (Fig. 3B). Based on observations that PFV IN is primarily monomeric in solution [4], [7] and that HIV-1 IN monomers can catalyze proficient concerted integration activity in the absence of LEDGF/p75 protein [32], there is some reason to believe that monomers serve as obligate intermediates in intasome assembly. Our results with MMTV and Rev-A INs are consistent with this hypothesis, as both proteins predominantly migrated as monomers on a gel filtration column (Fig. S1) and also preferentially catalyzed concerted integration activity *in vitro* (Fig. 3).

*Retroviridae* is classified into seven genera [33]. Except for epsilonretroviruses, integration site preferences are known for at least one member of each genus, which has further led to the classification of the viral INs into three clusters. Each cluster is characterized by integration site preference and length of target site duplication, as well as IN sequence phylogeny: (i) near transcription start sites and CpG islands, generation of a 4 bp duplication (the gammaretrovirus and spumavirus MLV and PFV, respectively); (ii) within genes or transcription units, 5 bp duplication (the lentiviruses); (iii) randomly dispersed, 6 bp duplication (alpharetrovirus avian sarcoma-leukosis virus, betaretrovirus MMTV, and deltaretrovirus human T-cell leukemia virus) [26], [34], [35]. According to this classification and knowing that MLV and Rev-A gammaretroviral INs are 40.4% identical and 59.6% similar at the amino acid level (Fig. S2), we would have predicted a 4 bp duplication of target DNA following concerted Rev-A integration, yet a 5 bp duplication was observed (Fig. 4A). Of note, the 5 bp duplication is consistent with that observed for SNV in cell culture [27], [28].

Based on prior work with PFV IN, we conjecture that the preference for particular bases at the sites of integration is dictated by IN-target DNA interactions [5]. Accordingly, both MLV and Rev-A appear to select for similar base contacts during integration (Fig. 4B). The key difference between these site preferences is that Rev-A IN yields the 5 bp (A/V)(T/H)W(A/D)(T/B) target site duplication where MLV IN generates the 4 bp VTAB duplication (Fig. 4B). Our results therefore clarify that the spacing of the cut in target DNA across the major groove is apparently more evolutionarily flexible than are the gammaretroviral IN-target DNA contacts during integration.

## Conclusions

From five initially studied novel retroviral IN proteins, Rev-A IN and MMTV IN were produced in reasonable yields from *E. coli*, and preferentially catalyzed concerted vDNA integration *in vitro*. Rev-A IN and MMTV IN have accordingly been selected for our structural biology pipeline. Our results also highlight that different viruses from the same retroviral genus (the gammaretroviruses in this case) can produce different sized duplications of host DNA sequence flanking their integrated proviruses. This information should be taken into account when using integration-specific parameters to classify different retroviruses into clusters or groups [26], [34], [35].

## Supporting Information

Figure S1 **Gel filtration chromatography analysis of purified MMTV and Rev-A IN proteins.** Based on the calibration curve calculated from the elution volumes of the noted globular protein standards, the predominant MMTV and Rev-A IN species migrated at ∼58 kDa and 60 kDa, respectively, while their calculated molecular weights are 35.6 kDa and 44.6 kDa, respectively. Vo, void volume; mAU, milli absorbance unit.
(TIF)

Figure S2 **Comparison of Rev-A and MLV IN proteins.** Alignment of MLV and Rev-A IN sequences generated using ESPript [37]. Red and yellow boxes indicate positions of amino acid identity and similarity, respectively.
(TIF)
