# Supplementary figures and images for: Biochemical Characterization of Novel Retroviral Integrase Proteins
Source: PLoS One. 2013 Oct 4;8(10):e76638. doi: 10.1371/journal.pone.0076638 (PMC3790719; doi:10.1371/journal.pone.0076638)

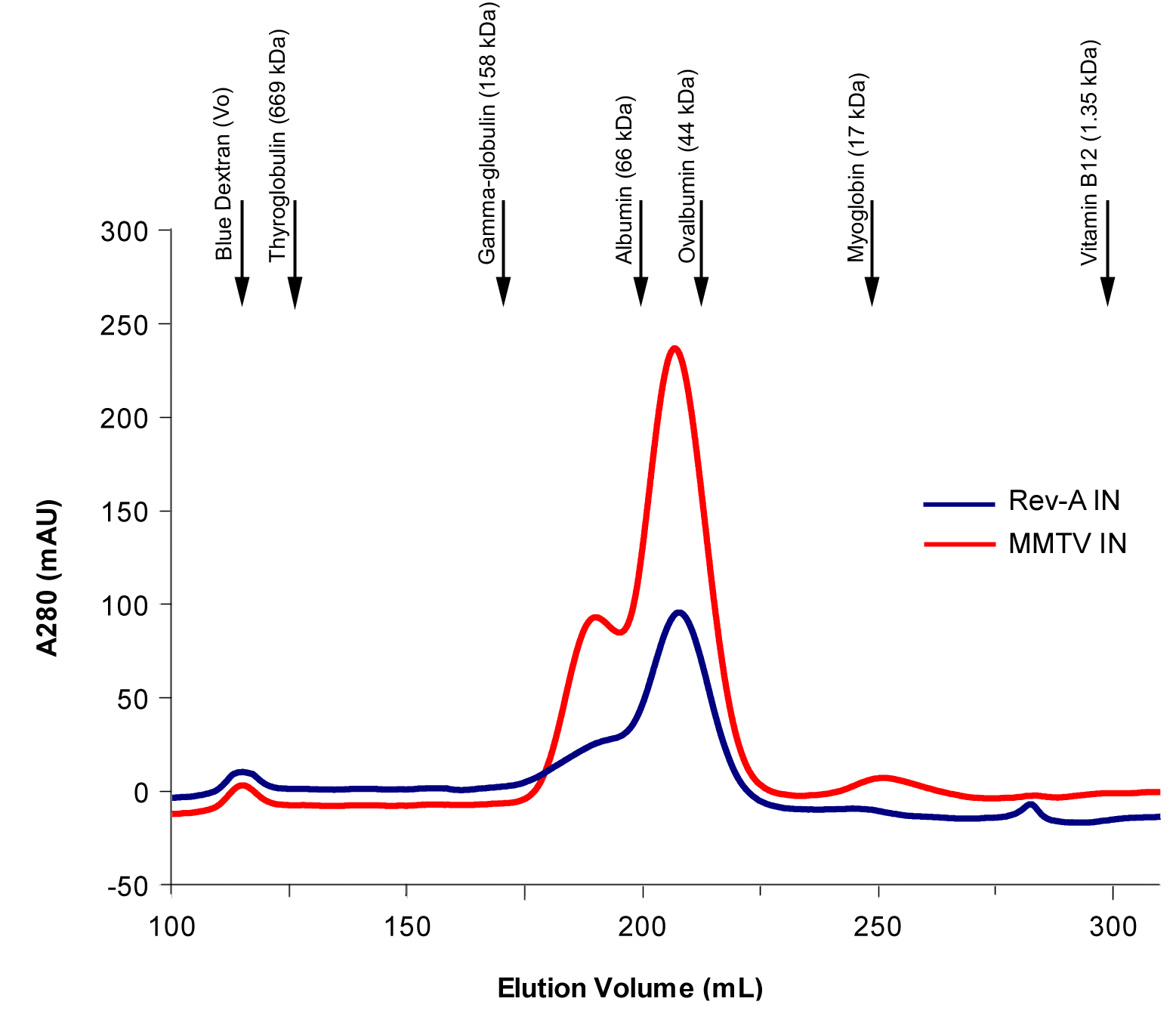

Supplement: Figure S1 — Gel filtration chromatography analysis of purified MMTV and Rev-A IN proteins. Based on the calibration curve calculated from the elution volumes of the noted globular protein standards, the predominant MMTV and Rev-A IN species migrated at ∼58 kDa and 60 kDa, respectively, while their calculated molecular weights are 35.6 kDa and 44.6 kDa, respectively. Vo, void volume; mAU, milli absorbance unit. (TIF) [file pone.0076638.s001.tif]

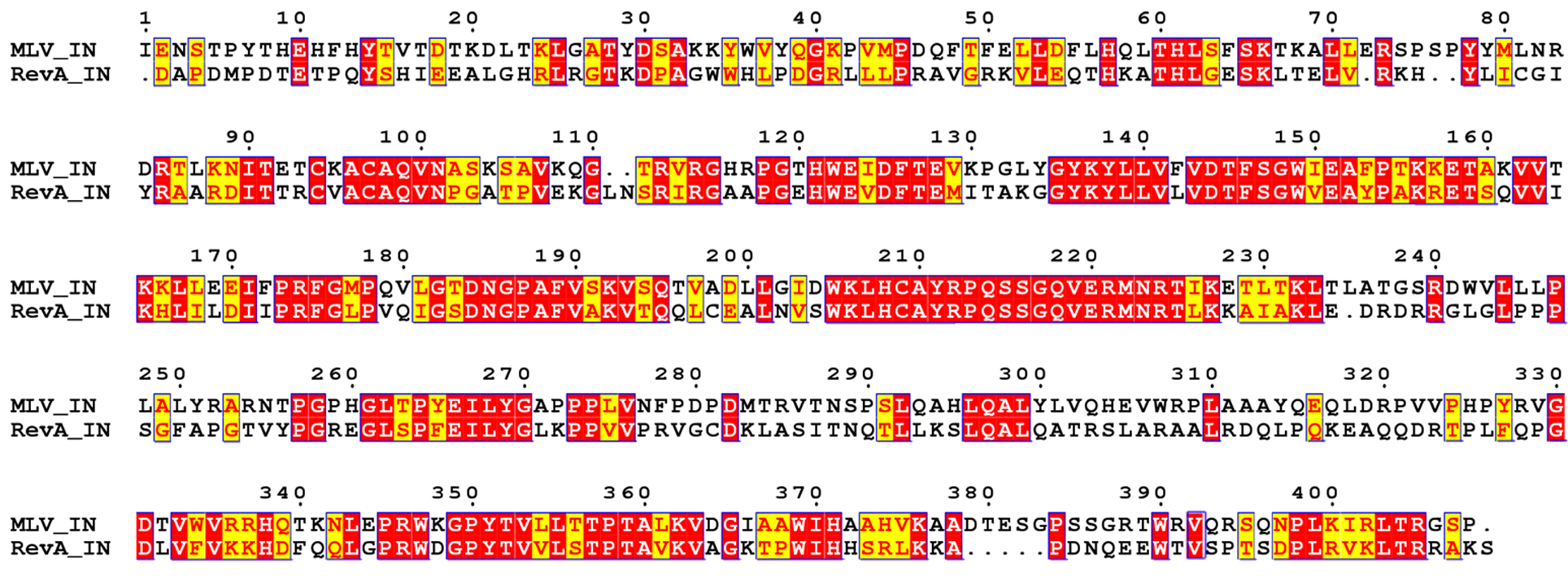

Supplement: Figure S2 — Comparison of Rev-A and MLV IN proteins. Alignment of MLV and Rev-A IN sequences generated using ESPript [37]. Red and yellow boxes indicate positions of amino acid identity and similarity, respectively. (TIF) [file pone.0076638.s002.tif]
